# Analysis of stereopsis and fusion in school-aged children with reduced visual acuity due to refractive error

**DOI:** 10.1371/journal.pone.0284112

**Published:** 2023-04-04

**Authors:** Hye Jun Joo, Dong Gyu Choi

**Affiliations:** Department of Ophthalmology, Kangnam Sacred Heart Hospital, Hallym University College of Medicine, Seoul, Korea; The Ohio State University, UNITED STATES

## Abstract

**Purpose:**

In patients with strabismus, the stereopsis and Worth 4-dot (W4d) tests have often been used to evaluate whether sensory fusion is achieved. However, if patients face difficulties undergoing the Titmus or W4d test because of poor visual acuity (VA) due to refractive error abnormalities, the results of these tests cannot be appropriately interpreted. Therefore, we evaluated the correlation between distance uncorrected VA (UCVA) and sensory status in children with reduced VA due to refractive error abnormalities to identify the effects of refractive errors on sensory test results.

**Methods:**

We retrospectively reviewed the medical records of 195 children with reduced VA with VA improvement ≥ 20/25, Titmus stereoacuity ≤ 50 arcsec, and fusion in the W4d result after refractive error correction with spectacles. We evaluated the correlation between distance UCVA in logMAR and sensory status measured by the near Titmus stereotest and distance W4d test. Additionally, the minimum required UCVA for interpreting Titmus or W4d results was assessed using a receiver operating characteristic (ROC) curve.

**Results:**

The UCVA showed a marginal but non-significant correlation with Titmus stereoacuity (p = 0.053) and a significant correlation with fusion in W4d (p < 0.001). The ROC curve analysis showed an optimal cut-point value of VA required for interpreting the results of W4d test as 0.3 logMAR (20/40 in Snellen acuity).

**Conclusions:**

Correcting refractive error in advance may help appropriately interpret sensory status in school-aged children with reduced VA due to refractive error abnormalities.

## Introduction

The stereopsis test is a simple and easy method to evaluate binocular dysfunction and has been widely used in various clinical settings [1, 2]. The stereopsis test has been suggested to be an ideal test for visual screening because all optical, neural, and motor components in both eyes must be in working condition to achieve a normal stereoacuity threshold [2]. According to Levy and Glick [3], a linear relationship exists between the polaroid vectographic test of stereopsis with a given level of Snellen visual acuity (VA). Similarly, Donzis, Rappazzo, Burde, and Gordon [4] reported that the Titmus stereotest tended to improve with improvements in VA. On the other hand, Lam et al [5]. evaluated the range and variability of ophthalmological parameters such as VA, refraction, and stereoacuity in “normal” children classified by Gold Standard Ophthalmological examination. A wide range of scores on Randot testing was found in “normal” children, thus raising doubts on the value of the stereoacuity test as a screening device for binocular dysfunction. In addition, a preschool vision study by Amigo [6] concluded that children under 4 years old showed low sensitivity in the Titmus stereopsis test; therefore, it was unsuitable for vision screening in this age group.

The Worth 4-dot (W4d) test evaluates binocular fusion/suppression status. However, because the eyes are easily dissociated with red–green glasses in the W4d test, a patient with unstable but functionally useful binocular vision may exhibit a suppression response [7]. Moreover, in the distant W4d test at 5−6 m, binocular fusion may not be achieved in patients with poor VA due to refractive error even though they have the potential to achieve binocular fusion when refractive errors are corrected.

In patients with strabismus, the stereopsis and W4d tests have often been used to evaluate whether sensory fusion is achieved before and after non-surgical or surgical management [8, 9]. However, if patients face difficulties in undergoing the Titmus or W4d test because of poor VA due to refractive error abnormalities, the results of these tests cannot be appropriately interpreted.

The standard procedures for sensory test such as Titmus stereotest and W4d test are to be performed after appropriate full spectacle correction. However, in our clinic, these measurements have been made prior to and after refractive error correction. Therefore, in this study, we evaluated the sensory status by using the near Titmus stereotest and distance W4d test in children with reduced VA due to refractive error to determine the correlation between uncorrected VA (UCVA) and the sensory status. We also evaluated the optimal cut-point value of distance UCVA required for interpreting the results of the Titmus stereotest or W4d test in these subjects.

## Materials and methods

### Study design and subjects

We retrospectively reviewed the medical records of 195 children who visited the Pediatric Ophthalmology Clinic of Kangnam Sacred Heart Hospital for reduced VA between January 2015 and December 2019; successfully underwent the W4d test and Titmus stereotest from the first visit; and had a best-corrected VA (BCVA) of 20/25 or better, stereoacuity of 50 arc of seconds or better in the Titmus stereotest, and W4d result to fusion after refractive error correction with spectacles. The exclusion criteria were (1) any type of intermittent or constant strabismus, (2) corneal or retinal disease (3), prior history of ocular surgery, and (4) low cooperation in the Titmus stereotest and W4d test. All patients underwent unilateral cover test and alternate prism cover test to rule out strabismus. Slit-lamp examination and fundus examination were performed in all patients.

### Institutional Review Board statement

This study’s protocol adhered to the Declaration of Helsinki, and the requirement for informed consent was waived by the Institutional Review Board of Hallym University Medical Center (approval no. 2021-03-028) owing to the retrospective nature of the study.

### Measurement of VA

VA with and without wearing spectacles was measured using a Snellen VA chart at a distance of 5 m. BCVA was measured on the basis of the results of cycloplegic refraction with 1% cyclopentolate chloride (Cyclogyl, Alcon Lab. Inc., Fort Worth, TX, USA) and 1% tropicamide (Mydriacyl, Alcon Lab. Inc.). Snellen acuity was converted to logMAR equivalent for the analysis. The worse monocular UCVA was used for statistical analysis.

### Measurement of sensory status

Sensory testing using the Titmus stereotest at near (40 cm) and the W4d test at distance (5 m) was performed before and after wearing spectacles. The Titmus stereotest (Stereo Optical Co., Chicago, IL, USA) was performed while wearing polarized glasses. There are three separate tests (the fly, animals, and circles) that test stereoacuity down to 40 arcsec. During the W4d test, the patients wore red–green spectacles. The following are the possible results: (1) fusion if four lights are seen, (2) suppression if two or three lights are seen, and (3) diplopia if five lights are seen.

### Main outcome measures

The correlations of distance UCVA and sensory status measured by the near Titmus stereotest and distance W4d test were evaluated, respectively. The correlations between sensory status and several clinical variables, such as gender, age, and refractive errors (hyperopia and myopia in SE, astigmatism, and anisometropia), were also evaluated. The SE was defined as the dioptre (D) value of the sphere plus that of astigmatism /2. Anisometropia was defined as the difference in SE between the two eyes of >1D. For the analysis, the myopia group was defined as an SE of ≤−1.0D, hyperopia as an SE of ≥ +1.0D [10], and astigmatism as an absolute value of astigmatism of ≥ 0.50D. The optimal cut-point value of distance UCVA required for interpreting the results of the Titmus stereotest or W4d test as an indicator of sensory status before refractive correction in subjects with reduced VA due to refractive error was assessed using Younden’s index of receiver operating characteristic (ROC) curves.

### Statistical analyses

The relationship between the Titmus stereotest results and the clinical variables was analyzed using Spearman’s correlation anaylsis and Mann–Whitney U test. The correlation between fusion in the W4d test and clinical variables was analyzed using logistic regression analysis. The ROC curve was calculated to determine the optimal cut-point value of distance UCVA required for interpreting the results of the Titmus stereotest and the W4d test. The value of the Titmus stereotest was transformed to logarithmic units, and the eye with the worse VA value and refractive error was used for the analysis. Statistical significance was set at p < 0.05. All statistical analyses were performed using MedCalc® Statistical Software version 19.5.6 (MedCalc Software Ltd, Ostend, Belgium; https://www.medcalc.org).

## Results

A total of 195 subjects (102 boys and 93 girls) were enrolled in this study. The demographic data of the subjects are presented in Table 1. The mean age of the enrolled children was 8.81 ± 2.05 years (range: 5 to 15), and the mean distance UCVA was 0.30 ± 0.16 logMAR (range: 0.05 to 0.70 logMAR). The mean refractive error in SE was +2.21 ± 1.48D (range: +1.00 to +7.00D) in the hyperopia group and −1.77 ± 0.56D (range: −1.00 to −3.75D) in the myopia group. The mean absolute value of astigmatism was 1.48 ± 1.14D (range: 0.5 to 6.00D).

**Table 1:** Demographic characteristics of the enrolled subjects (N = 195).

Characteristics	Results
**Gender (male/female)**	102 (52%)/93 (48%)
**Age**	8.81 ± 2.05 [5 to 15]
**Myopia (n = 155)**	−1.77 ± 0.56 [−1.00 to −3.75]
**Hyperopia (n = 19)**	+2.21 ± 1.48 [+1.00 to +7.00]
**Astigmatism (n = 138)**	1.48 ± +1.14 [0.50 to +6.00]
**Anisometropia**	37 (19%)

Values are presented as numbers (%) or mean ± standard deviation (range). Myopia: spherical equivalent (SE) ≤ −1.0D; hyperopia: SE ≥ +1.0D; astigmatism: absolute value of astigmatism ≥ 0.50D; anisometropia: SE difference between the two eyes > 1.0D.

The correlation between the Titmus stereotest and clinical variables is listed in Table 2. There was a marginal but non-significant correlation between distance UCVA and the Titmus stereotest (p = 0.053). Younger age (p = 0.01), astigmatism (p = 0.008), and anisometropia (p = 0.008) were associated with poor stereoacuity. However, no significant correlation was noted between hyperopia and myopia in SE and stereoacuity. In addition, the assessment of a cut-point value of distance UCVA for interpreting stereopsis with the ROC curve failed because the UCVA did not show a statistically significant correlation with the results of the Titmus stereotest.

**Table 2:** Correlation between Titmus stereotest (log seconds) and clinical variables.

Variables	P-value
**Age**	0.01 *
**Gender**	0.562 †
**UCVA (logMAR)**	0.053*
**Myopia (n = 155)**	0.722*
**Hyperopia (n = 19)**	0.125*
**Astigmatism (n = 138)**	0.008†
**Anisometropia**	0.008†

*Spearman correlation analysis

†Mann–Whitney U test

UCVA: uncorrected visual acuity, myopia: spherical equivalent (SE) ≤ −1.0D; hyperopia: SE ≥ +1.0D; astigmatism: absolute value of astigmatism ≥ 0.50D; anisometropia: SE difference between the two eyes > 1D.

Table 3 shows the analysis of the association between fusion in the W4d test and clinical variables. Among 195 children, 108 children (55.4%) showed “fusion” before wearing spectacles, whereas 87 (44.6%) failed to achieve “fusion.” Logistic regression analysis revealed that worse UCVA (p < 0.001), larger absolute value of myopia in SE (p = 0.001), and younger age (p = 0.019) were negatively correlated with fusion in the W4d test.

**Table 3:** Logistic regression analysis of the presence (n = 108) and failure (n = 87) of fusion in the W4d test and clinical variables (N = 195).

Variables	P-value
**Age**	0.019
**Gender**	0.062
**UCVA (logMAR)**	<0.001
**Myopia (n = 155)**	0.001
**Hyperopia (n = 19)**	0.690
**Astigmatism (n = 138)**	0.98
**Anisometropia**	0.202

W4d: Worth 4-dot; UCVA: uncorrected visual acuity; myopia: spherical equivalent (SE) ≤ −1.0D; hyperopia: SE ≥ +1.0D; astigmatism: absolute value of astigmatism ≥ 0.50D; anisometropia: SE difference between the two eyes > 1.0D.

**Fig 1:**
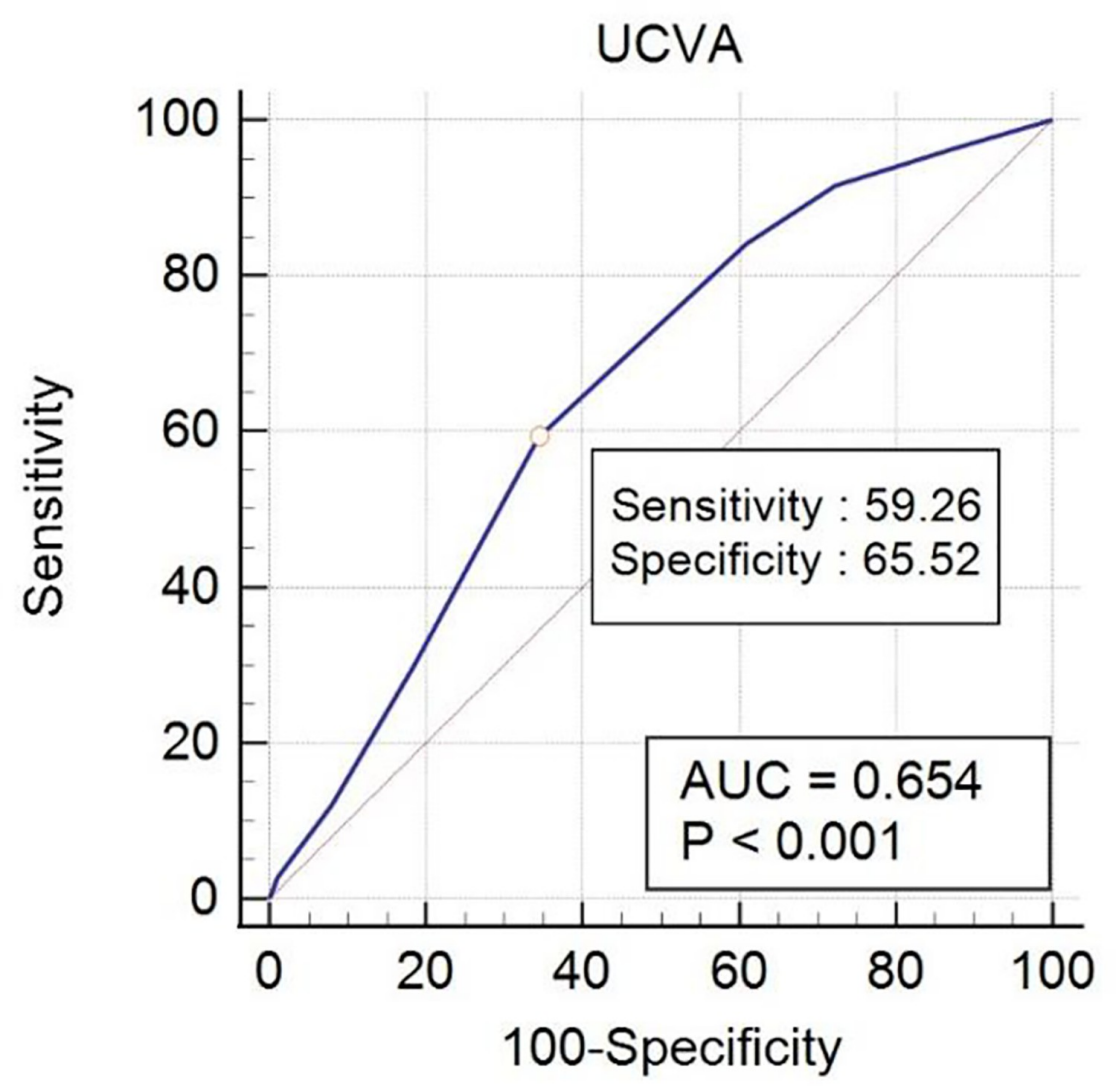
Receiver operating characteristic (ROC) curve analysis revealing the relationship between distance uncorrected visual acuity (UCVA) and the Worth 4-dot (W4d) test. ROC curve analysis identified a UCVA value of 0.3 logMAR (20/40 in Snellen acuity) or better as the optimal cut-point value of visual acuity required for interpreting the results of the W4d test (ROC area under the curve: 0.654; 95% CI: 0.582−0.720; p < 0.001). A UCVA value of 0.3 logMAR (20/40 in Snellen acuity) or better yielded a sensitivity of 59.26% and a specificity of 65.52%.

ROC curve analysis identified a UCVA value of 0.3 logMAR (20/40 in Snellen acuity) or better as the optimal cut-point value of VA required for interpreting the results of the W4d test. In the ROC curve analysis using a UCVA value of 0.3 logMAR (20/40 in Snellen acuity) or better, the W4d test predicted fusion with a sensitivity of 59.26% and a specificity of 65.52% (ROC area under the curve: 0.654; 95% CI: 0.582−0.720; p < 0.001) (Fig 1).

## Discussion

Stereoacuity refers to the smallest amount of horizontal retinal image disparity (measured in arcsec) giving rise to the perception of relative depth or stereopsis [11]. Stereoscopic resolution depends on the VA. A general guide on the effect of image blur (Snellen VA) on stereoacuity is as follows: 40 arcsec corresponding to 20/25, 60 arcsec to 20/40, 100 arcsec to 20/60, 200 arcsec to 20/80, and 800 arcsec to 20/200 [12]. Recently, Hrisos et al. [13] investigated the influence of unilateral visual impairment on stereoacuity and neurodevelopmental functioning in preschool children and concluded that impaired VA is moderately correlated with reduced stereoacuity. Lam et al. [5] reported the random-wide distribution of stereoacuity among completely normal children, thus questioning the level of normal stereopsis. Similarly, Fisher [14] demonstrated that high degrees of binocular cooperation may be present in patients with high, deficient, or even absent stereoacuity. The current study demonstrated that there was no significant correlation between the Titmus stereotest and distance UCVA and the degree of myopia, and this result corresponds to that of Lam et al. [5] and Yang, et al [15]. Given that the Titmus stereotest is measured at a near distance (33 cm), visual blurring due to refractive errors may be overcome with accommodative power in hyperopes and small myopes to obtain good stereopsis.

Increased astigmatism was found to significantly correlate with degraded stereopsis. This result is consistent with several previous studies. Chen et al. [16] concluded that depth discrimination degrades with increasing degrees of astigmatic blur whether induced monocularly or binocularly. Previous studies on adults reported a reduction in depth discrimination in monocularly induced astigmatic blur [17, 18]. The current study showed that uncorrected astigmatism in children also limits binocular stereopsis, and this result is similar to those shown in adults.

Brooks et al. [18] showed that anisometropia, both spherical and astigmatic, can have a potentially significant adverse effect on high-grade binocular interaction in stereoacuity and W4d test. They postulated that the mechanisms underlying the loss of binocularity seem to involve foveal suppression. However, the current study revealed that anisometropia did not show a statistical correlation with W4d fusion, whereas it was significantly correlated with stereoacuity. Therefore, the deterioration of binocularity in anisometropia may not be caused by foveal suppression but by optical blur because the subjects in this study showed normal binocularity and VA after refractive error correction.

According to the ROC curve for the W4d test at distance (5 m), a UCVA value of 0.3 logMAR (20/40 in Snellen acuity) or better provides convincing sensitivity and specificity for the interpretation of the W4d test in various causes of sensory anomalies, such as strabismus or monofixation syndrome, which causes foveal suppression in subjects with refractive error abnormalities before glasses wearing. However, because the sensitivity and specificity were not high enough, the result should be considered in conjunction with other clinical findings. Older individuals showed better results in the Titmus stereotest and W4d test, with statistical significance. The Titmus stereotest possess monocular clues in low-grade stereograms [19], where age may affect the result.

There are some limitations to our study. First, we included subjects who underwent complete ophthalmologic examinations including cycloplegic refraction, ocular motility, and fundus examinations and had “normal” findings, except for refractive errors. However, there is a small possibility that microstrabismus was not detected in this study by using the unilateral cover test and alternate cover test. Second, the amplitude of accommodation was not routinely measured in subjects who showed “normal” ophthalmologic findings, except for refractive errors, thus possibly causing a minor bias for the analysis. Third, Younden’s index from the ROC curve of the correlation between UCVA and W4d result was not high enough, therefore, the reuslt from ROC curve should be considered in conjunction with other clinical findings. Lastly, given that near VA has not been routinely measured in our clinic, we should analyze the relation and Titmus stereotest (at 40 cm) and distance VA instead of near VA in this retrospective study. Further prospective studies with standardized designs including near VA measurement is needed to collect sufficient data for the statistical analyses in the future.

Nevertheless, our study is meaningful in several aspects. Most studies in this area focused on adults and speculated that similar results will be observed in children. Moreover, most previous studies inferred results via experimentally induced anisometropia in normal subjects [3, 4, 16–18]. On the other hand, the current research was based on children that have actual refractive errors. Finally, many studies have investigated the relationship between stereoacuity test and refractive errors; however, there are only few studies on the W4d test. We found a significant negative correlation between distance UCVA and fusion in the W4d test, and the ROC curve suggested that refractive correction should be performed before performing the W4d test in patients with low VA due to refractive error. Although our results cannot be used as a definitive standard for interpreting sensory tests, these should be taken into consideration when developing such guidelines. We hope that further studies will be undertaken to fully evaluate this issue.

In conclusion, even in children with full potential for stereopsis and fusion, the fusion rate may be low in children with reduced VA. Therefore, if a patient with reduced VA due to any cause demonstrates decreased fusion and stereopsis, we cannot conclude that the fusion is truely reduced. Refractive error correction should be performed before evaluating sensory status in children with reduced VA due to refractive error.

## Supporting information

S1 Data (XLSX)
